# J’Accuse….. Or The Plight of pro-bono Volunteer Scientists in Academic Publishing

**DOI:** 10.1186/s40246-022-00413-z

**Published:** 2022-09-28

**Authors:** Juergen K. V. Reichardt, George P. Patrinos, Poh San Lai, Giuseppe Novelli

**Affiliations:** 1 Australian Institute of Tropical Health and Medicine, James Cook University, Cairns, Australia; 2 Department of Pharmacy, University of Patras School of Health Sciences, Patras, Greece; 3 Department of Paediatrics, Yong Loo Lin School of Medicine, National University of Singapore, Singapore, 119228 Singapore; 4 Department of Biomedicine and Prevention, Tor Vergata University of Rome, Rome, Italy

The immortal words of Èmile Zola: “J’accuse…” were his introduction to a letter on the mistreatment of a French citizen [1]. We believe Springer Nature (and other commercial publishing houses) need to similarly clean up their acts in the area of pro-bono volunteer scientists and hence this melodramatic yet appropriate start to this letter.

Many academics volunteer and devote their limited time as referees and on editorial boards for commercial, i.e., for-profit, publishing houses, including Springer Nature. This scenario applies prolifically to all authors specifically who serve as frequent referees—in many manuscript-handling electronic systems “author” and “referee” are interrelated terms—on editorial boards and more. As academics, we usually view these activities as part of our service to the scientific community. However, commercial publishing houses, including Springer Nature, are for-profit corporations to which we are providing a free service as unpaid volunteer workers. Nonetheless, highly skilled but unpaid volunteer workers should neither be taken for granted nor forgotten altogether.

Recently, Springer Nature switched some of its journals, for several of us as Associate Editors specifically at *Human Genomics,* but supposedly also numerous other publications, to an in-house editorial platform: SNAPP (Springer Nature's Article Processing Platform). Unfortunately, this was done without consultation and resulted in a tool that is not even close to fit-for-purpose forced upon users. This particular situation raised broader issues on the role of unpaid volunteers at for-profit corporations in scientific publishing. Unpaid volunteer workers keep commercial publishing houses in business through their peer review and editorial work and deserve respectful treatment, including specifically appropriate consultations and fit-for-purpose tools. Springer Nature has dismally failed with SNAPP, but this unfortunate situation now offers the opportunity for a reset in the relationship of unpaid workers and Springer Nature specifically but also in other for-profit publishing houses more generally.

We are suggesting that this unpleasant specific situation affords Springer Nature and *Human Genomics* specifically the opportunity and catalyst to highlight the issue of unpaid volunteer scientists in peer-reviewed scientific publishing such as this prestigious journal. Specifically, we are calling on Springer Nature to develop an industry-leading respectful and comprehensive policy for unpaid volunteer workers, including appropriate consultations and fit-for-purpose tools. At the same time, we propose that Springer Nature establishes a rewarding system for referees and associate editors, perhaps along the lines of the microattribution concept that exists for authors [2] to get credit for their contribution to scientific society but without compromising the confidentiality and robustness of the peer review process. The evolving peer review landscape such as open peer review and the emergence of ORCID and Publons services do not fully address the ethical duty of Publishers to value and credit the free academic labor of referees and editors. While payment for peer review [3] has been suggested as a solution to this cumbersome and time-consuming academic undertaking by highly skilled workers [4], there should be more open conversations on the issue of respectful treatment and valuing of referees and editors. We believe that it is time for a mindset reset by publishing houses before more reviewers and editors “go on strike.” Furthermore, we are recommending that this new policy be widely publicized and specifically also published broadly. Other publishing houses may well wish to follow suit in appreciating unpaid volunteer workers in commercial publishing.
